# Application of the Full-Width-at-Half-Maximum Image Segmentation Method to Analyse Retinal Vascular Changes in Patients with Diabetic Retinopathy

**DOI:** 10.1155/2022/6726499

**Published:** 2022-08-08

**Authors:** Bo Lun Xu, Yi Jie Li, Wen Li Zhou, Hai Jing Zhan, Jie Yi Lu, Yu Hua Tong

**Affiliations:** ^1^The Quzhou Affiliated Hospital of Wenzhou Medical University, Quzhou People's Hospital, Quzhou, Zhejiang, China; ^2^The First Affiliated Hospital of Jiangxi Medical College, Shangrao, Jiangxi, China; ^3^Shanghai Sixth People's Hospital, Shanghai, China; ^4^The Second Clinical Medical College, Zhejiang Chinese Medical University, Hangzhou, China

## Abstract

This study used spectral domain optical coherence tomography (SD-OCT) and full-width-at-half-maximum image segmentation to investigate the morphological changes of retinal blood vessels in patients with diabetic retinopathy (DR). Seventy-five patients with type 2 diabetes mellitus (T2DM) without DR and 65 patients with DR were studied. Vascular images of superior temporal region B of the retina were obtained by SD-OCT. The edges of retinal vessels were identified by the full-width-at-half-maximum image segmentation method. The lumen diameter, wall thickness (WT), wall cross-sectional area (WCSA), and wall-to-lumen ratio (WLR) were investigated. We found that, compared with the no diabetic retinopathy (NDR) group, patients in the DR group had an increased retinal arteriolar lumen diameter (RALD), retinal arteriolar outer diameter (RAOD), and WT (128.80 *μ*m versus 104.88 *μ*m; 147.01 *μ*m versus 135.60 *μ*m; 18.29 *μ*m versus 15.26 *μ*m, *P* < 0.05, respectively). The retinal venular lumen diameter (RVLD), retinal venular outer diameter (RVOD), and venular WT in the DR group were also increased (146.17 *μ*m versus 133.66 *μ*m; 180.20 *μ*m versus 156.43 *μ*m; 17.01 *μ*m versus 11.38 *μ*m, *P* < 0.05, respectively). The morphological changes in retinal vessels were significantly correlated with DR stage. In conclusion, in diabetic patients with DR, both retinal arteries and veins are widened and exhibit increased vascular thickness.

## 1. Introduction

Diabetic retinopathy (DR) is a very common chronic complication of diabetes. Approximately 30% of diabetic patients have DR lesions, and approximately 30% of DR patients will suffer from visual loss due to severe retinopathy. The data from the Early Treatment Diabetic Retinopathy Study research group and the Diabetic Retinopathy Study research group showed that regular follow-up, necessary and appropriate vitreous surgery, retinal laser photocoagulation, and other interventions can reduce the risk of severe vision loss in 90% of DR patients. If patients with proliferative DR can be evaluated and treated early, the blindness rate of DR will be significantly reduced. The results of a study in the United States showed that this approach can reduce the blindness rate from 50% to less than 5%. Therefore, early detection of DR and timely and effective early treatment are key to reducing the incidence of visual disability in diabetic patients. Regular DR screening for diabetic patients is an effective measure to reduce blindness caused by diabetes.

DR is mainly a fundus change due to diabetes-induced retinal microangiopathy. Insufficient oxygen supply to retinal vessels and the local inflammatory response are the pathological mechanisms underlying the development of DR, and these changes in the intraocular microenvironment can lead to compensatory dilation of retinal blood vessels. For example, irregular dilation of retinal veins (venous beading) is a typical clinical feature of severe DR. During DR pathogenesis or DR progression, in addition to the risk factors such as glycosylated haemoglobin concentration, blood pressure, and duration of diabetes, recent epidemiological studies have also shown that retinal vasodilatation is a risk factor for the progression of DR patients. The dilation of retinal veins is a high-risk factor for the pathogenesis of DR, which may be related to the dysfunction of retinal vascular endothelial cells, changes in the levels of inflammatory factors, and hyperglycaemia, and these factors all play important roles in the pathogenesis and progression of DR.

In this study, DR, a common eye disease that seriously threatens the quality of human life, was studied. We quantitatively analysed the lumen diameter (LD) of retinal large vessels in patients with DR by spectral domain optical coherence tomography (SD-OCT) and explored the correlation between the LD of retinal large vessels and the pathogenesis and progression of DR in order to provide an accurate, objective, and quantitative method for the early detection and monitoring of DR.

## 2. Methods

### 2.1. General Data and Grouping

The study subjects were randomly selected from patients who visited the outpatient clinic of Quzhou People's Hospital from January 2016 to November 2017. The study was approved by the Research Ethics Committee of Quzhou People's Hospital and was conducted in adherence to the tenets of the Declaration of Helsinki. Informed consent was obtained from all participants. Enrolment criteria for the experimental group were as follows: patients above 50 years of age who were diagnosed with type 2 diabetes mellitus (T2DM) in the Department of Endocrinology, the patients or their family members who could clearly describe a history of diabetes and their diagnostic and treatment process, and patients who could cooperate with fundus examination after cycloplegia. Exclusion criteria were as follows: patients with other types of diabetes, hypertension, and previous or current cardiovascular disease (excluding cerebrovascular disease), myopia exceeding −6.0 D, severe refractive medium opacity, intraocular pressure higher than 21 mmHg, history of glaucoma, ocular ischaemic syndrome, uveitis, ocular space-occupying lesions, retinal disease, or previous laser or phacoemulsification surgery, rheumatic immune disease, pulmonary heart disease, severe carotid artery stenosis, nephropathy, or a history of smoking and drinking. A total of 140 patients (58 females and 82 males) were included. The patients were divided into a no DR (NDR) group (75 patients) and a DR group (65 patients) according to the presence or absence of DR.

### 2.2. Experimental Procedures

#### 2.2.1. Acquisition of OCT Images of Retinal Blood Vessels

**Figure 1:**
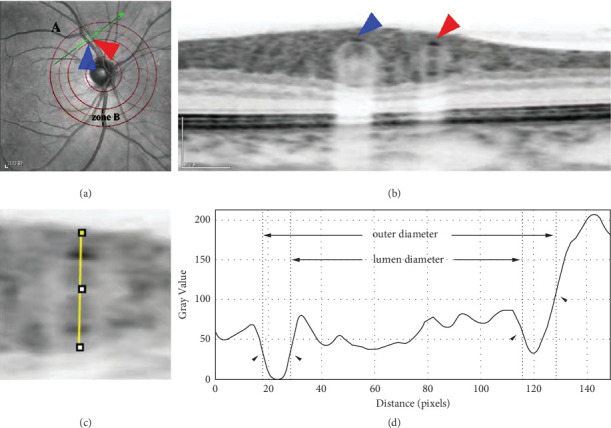
Schematic diagram of measuring retinal blood vessels on OCT images using the FWHM method. (a) Schematic diagram of region B positioning. The scanning line runs perpendicular to the axis of a retinal vessel in region B with the red arrow indicating the artery and the blue arrow indicating the vein. (b) The OCT image clearly showing the cross-sections of the retinal artery (red arrow) and the retinal vein (blue arrow). (c) A line through the centre of the circle drawn between the upper and lower walls of a vessel to produce a greyscale density curve of the vessel wall. (d) The boundaries of the upper and lower walls of the vessel determined with the FWHM segmentation method (shown by arrows), and the luminal and outer diameters of the vessel are calculated after the boundaries are determined.

All OCT images were acquired with an SD-OCT instrument (Heidelberg Engineering, Heidelberg, Germany). The subject was asked to sit down, place the head on the examination table, and look at the blue light inside the lens. The retinal artery and its accompanying veins in superior temporal region B of the right eye were scanned with an SD-OCT instrument. Region B was defined as the superior temporal region at a distance of 0.5 to 1.0 disc diameter from the optic disc edge (Figure 1(a)). First, in terms of morphology, the characteristics of the blood vessels in region B were more consistent with the description of arterioles and venules. Second, after the retinal blood vessels emanate from the centre of the optic disc, arteriovenous crossing and pulsation of the retinal arteries were less frequent in the region B, a certain distance from the optic disc, which did not affect the measurement of the diameters of the retinal artery or veins. The scanning line was adjusted to be as nearly perpendicular to the vascular axis as possible. If the vessel had branched before reaching region B, then the part before branching was scanned. After scanning, the vertical-to-horizontal ratio of the obtained OCT images was first adjusted to 1 : 1 *μ*m, the image was magnified to 800%, and then the OCT image was saved in BMP format. Only the images that clearly showed the vessel wall were used for the following analysis. At least five clearly scanned OCT images of every vessel were taken for analysis.

#### 2.2.2. Methods of Measuring Retinal Vessels

The full-width-at-half-maximum (FWHM) method was used to measure the LD, outer diameter (OD), and blood vessel wall thickness (WT) of the retinal blood vessels in the OCT images. The OCT images in BMP format were opened in the ImageJ software (National Institutes of Health), and the line tool was used to draw a vertical line through the middle of the blood vessel to obtain the density curve. There were two concave-upward curves on the density plot, representing the upper and lower walls of the vessel in the OCT image. On the left and right sides of each curve, the maximum and minimum values of the crest and trough were determined using the average of three consecutive values, and the median value between the maximum and minimum values was calculated. On each side of the curve, a linear function was fitted with continuous points with the greatest difference, and the intersection of this linear function with the horizontal line of the median value was the position of the edge. Finally, ImageJ software automatically identified the distance between the boundary points of the two curves and calculated the LDs and ODs of the retinal vessels (Figure 1).

All procedures were performed by the same experienced ophthalmologist. Because there is a good correlation between the measured retinal vessel diameters of the left eye and those of the right eye, only the retinal vessels in the right eye were measured in each patient. Each vessel of all subjects was measured three times with the FWHM method, and the average value was taken to obtain the retinal arteriolar lumen diameter (RALD), retinal arteriolar outer diameter (RAOD), retinal venular lumen diameter (RVLD), and retinal venular outer diameter (RVOD). Other morphological parameters of retinal vessels were calculated as described [1]. The formulas for the retinal artery are as follows: WT = (RAOD − RALD)/2; WLR = (RAOD − RALD)/RALD; WCSA = 3.14 × (RAOD^2^ − RALD^2^)/4. The formulas for the retinal vein were the same as those of the artery, and the corresponding venous values could be substituted into the formula. The results of the NDR group and DR group were compared.

#### 2.2.3. Statistical Analytical Methods

SPSS 21.0 was used. Measurement data are expressed as *x* ± *s*. Student's *t*-test was used to compare the retinal vessel diameters between the two groups. The *χ*^2^ test was used to compare count data between the groups. Univariate analysis was performed to detect correlations between the structural parameters (LD and the OD, WLR, WT, and WCSA of retinal blood vessels) and other parameters (age, DR stage, and duration of diabetes). Multivariate linear regression was performed with the LD, OD, WLR, WT, and WCSA of retinal blood vessels as dependent variables and the DR stage, duration of diabetes, and age as independent variables. *P* values <0.05 were considered statistically significant.

#### 2.2.4. Inspection Items

Fundus examination was performed under cycloplegia to determine the stage of DR, and the patient's history of systemic diseases and ocular diseases was collected.

#### 2.2.5. DR Staging

The stage of DR was determined according to the staging criteria in the 2014 Chinese Guideline for Clinical Diagnosis and Treatment of Diabetic Retinopathy. Stage I was the mild nonproliferative phase, stage II was the moderate nonproliferative phase, stage III was the severe nonproliferative phase, stage IV was the early proliferative phase, stage V was the fibroproliferative phase, and stage VI was the late proliferative phase.

## 3. Results

### 3.1. Comparison of General Data (Table 1)

In the NDR group there were 39 males and 36 females, and in the DR group there were 43 males and 22 females (*χ*^2^ test *P* > 0.05). The independent-samples *t*-test was used for the comparison of age (NDR: 62.40 ± 10.05; DR: 65.50 ± 8.41) and body mass index (NDR: 25.26 ± 3.32; DR: 24.30 ± 3.32), and the differences were not significant (both *P* > 0.05).

#### 3.1.1. Disease Staging of DR Patients

In the DR group, 37 patients had stage I DR, 11 patients had stage II DR, 11 patients had stage III DR, four patients had stage IV DR, and two patients had stage V DR.

### 3.2. Comparison of Retinal Arteries between the NDR and DR Groups (Table 2)

The RALD, RAOD, and WT of the NDR group were 104.88 ± 15.68 *μ*m, 135.60 ± 17.22 *μ*m, and 15.26 ± 2.56 *μ*m, compared to 128.80 ± 36.00 *μ*m, 147.01 ± 17.55 *μ*m, and 18.29 ± 5.97 *μ*m in the DR group (*t* = −5.214, *P* = 0.000; *t* = −3.872, *P*=0.000; *t* = −3.998, *P*=0.000, respectively). The WLR of the NDR group (0.29 ± 0.06) was larger than that of the DR group (0.28 ± 0.05), but the difference was not significant (*t* = 1.756, *P*=0.081). The WCSA (5685.20 ± 1255.26 *μ*m^2^) of the NDR group was significantly smaller than that (6647.51 ± 1590.52 *μ*m^2^) of the DR group (*t* = −3.333, *P*=0.001).

### 3.3. Comparison of Retinal Veins between the NDR and DR Groups (Table 3)

The RVLD, RVOD, and WT of the NDR group were 133.66 ± 21.07 *μ*m, 156.43 ± 23.36 *μ*m, and 11.38 ± 2.33 *μ*m, respectively, compared to 146.17 ± 34.42 *μ*m, 180.20 ± 38.70 *μ*m, and 17.01 ± 4.23 *μ*m in the DR group (*t* = −2.545, *P*=0.012; *t* = −4.313, *P*=0.000; *t* = −9.529, *P*=0.000). The WLR (0.17 ± 0.03) in the NDR group was significantly smaller than that (0.23 ± 0.05) in the DR group (*t* = −7.827, *P*=0.000). The WCSA (5321.03 ± 1695.19 *μ*m^2^) in the NDR group was significantly smaller than that (8962.13 ± 3897.34 *μ*m^2^) in the DR group (*t* = −6.982, *P*=0.000).

### 3.4. Retinal Artery-Associated Factors (Table 4)

RALD was positively correlated with DR stage (*r* = 0.480; *P* < 0.001). WT was positively correlated with DR stage (*r* = 0.524; *P* < 0.001). WLR was positively correlated with age (*r* = 0.271; *P*=0.029). Other variables were not correlated.

### 3.5. Retinal Vein-Associated Factors (Table 5)

RVLD was positively correlated with DR stage (*r* = 0.588; *P* < 0.00); RVOD was positively correlated with DR stage (*r* = 0.603; *P* < 0.001). WT was positively correlated with DR stage (*r* = 0.369; *P*=0.003). WLR was positively correlated with diabetes duration (*r* = 0.280; *P*=0.024). WCSA was positively correlated with DR stage (*r* = 0.567; *P* < 0.001). Other variables were not correlated.

### 3.6. Multivariate Linear Regression Analysis of Retinal Artery Variables (Table 6)

Among the factors age, DR stage, and diabetes duration, only DR stage (*b* = 17.633, *P* < 0.001) had a linear relationship with RALD (*R*^2^ = 0.231, *F* = 18.908), and the regression equation was RALD = 82.963 + 17.633 × DR stage. There was a linear relationship between DR stage (*b* = 3.193, *P* < 0.001) and WT (*R*^2^ = 0.274, *F* = 23.817), and the regression equation was WT = 9.997 + 3.193 × DR stage. There was a linear relationship between age (*b* = 0.002, *P*=0.029) and WLR (*R*^2^ = 0.074, *F* = 5.006), and the regression equation was WLR = 0.162 + 0.002 × age.

### 3.7. Multivariate Linear Regression Analysis of Retinal Vein Variables (Table 7)

There was a linear relationship between DR stage (*b* = 33.287, *P* < 0.001) and RVLD (*R*^2^ = 0.346, *F* = 18.908), and the regression equation was RVLD = 92.532 + 20.632 × DR stage. There was a linear relationship between DR stage (*b* = 23.819, *P* < 0.001) and RVOD (*R*^2^ = 0.364, *F* = 36.012), and the regression equation was RVOD = 118.273 + 23.819 × DR stage. There was a linear relationship between DR stage (*b* = 1.593, *P*=0.003) and WT (*R*^2^ = 0.136, *F* = 9.916), and the regression equation was WT = 12.871 + 1.593 × DR stage. There was a linear relationship between diabetes duration (*b* = 0.004, *P*=0.024) and WLR (*R*^2^ = 0.078, *F* = 5.344), and the regression equation was WLR = 0.208 + 0.004 × diabetes duration. There was a linear relationship between DR stage (*b* = 2251.506, *P* < 0.001) and WCSA (*R*^2^ = 0.321, *F* = 29.814), and the regression equation was WCSA = 3108.217 + 2251.506 × DR stage.

## 4. Analysis and Discussion

Retinal vessels are the only blood vessels in human body that can be directly observed and measured. They are small blood vessels that are similar in structure and physiological function to the terminal blood vessels of important organs such as the heart, brain, and kidney. Therefore, the change in the diameters of retinal vessels can reflect the physiological and pathological changes in other organs, tissues, and their blood vessels to a certain extent, so their lesions have an important auxiliary role in the diagnosis of other systemic diseases.

Diabetes mellitus is a chronic metabolic disease characterized by hyperglycaemia. It can be complicated by chronic lesions of multiple organs such as eyes, kidneys, nerves, and blood vessels. DR will cause decreased visual acuity and even blindness in patients. Retinal vessels can be examined to study the relationship between changes in the morphological characteristics of retinal vessels and diabetes [2]. However, there are few methods of retinal vascular monitoring. The morphology of retinal vessels is often observed and followed up with the help of ophthalmoscopy, fluorescein angiography, colour fundus photography, and other means in clinical practice to track the changes in arterial and venous diameters, and tools for more accurate and objective quantitative detection are lacking. At present, many large institutions that conduct epidemiological investigations obtain retinal blood vessel-related parameters using fundus photography. In this study, semiautomatic blood vessel measurement software was used to obtain a fundus photograph centred on the optic disc. Blood vessels in the annular area of 0.5 D–1.0 D from the edge of the optic disc were imaged, and the six largest arterioles and venules were used to measure the retinal blood vessels. The relative central retinal artery equivalent (CRAE) and central retinal vein equivalent (CRVE) data were obtained by conversion. Recently, researchers reported the use of deep learning models to automatically measure retinal vessel diameters in fundus photographs [3]. Others utilize a system called EyeArt, a cloud-based automated AI eye screening technology that can effectively help endocrinologists, diabetologists, and general practitioners address the growing concerns regarding DR screening and monitoring [4].

SD-OCT is an important imaging examination system for retinal tomography analysis. It is mainly used for quantitative and qualitative analysis of retinal tissue structure. It has the advantages of clear imaging, high accuracy, capacity for dynamic continuous tomography analysis, and no invasive damage [5]. At the same time, SD-OCT has eye-tracking function and can lock onto the blood vessels to be measured during eye rotation. Therefore, it is suitable to be used as a tool for analysing retinal vascular structure and obtaining tomographic images of blood vessels, which are more intuitive and convenient and can accurately locate and repeat the detection. In recent years, microdensitometry has been widely used for such purposes as the measurement and evaluation of retinal blood vessels in fundus photography [6,7]. The more widely used method is the FWHM algorithm, which is a segmentation method based on the grey value of the image. It is often applied to the identification and measurement of the edges of CT and magnetic resonance images [8]. It can more quickly and stably determine blood vessel boundaries and is less sensitive to the interference of noise points and adjacent tissues [9]. Therefore, the difference and innovation in this study compared with other studies were that we used the FWHM segmentation method to obtain truly accurate LDs and ODs of retinal blood vessels on SD-OCT images. Additionally, other structural values, such as WT, wall cross-sectional area (WCSA), and wall-to-lumen ratio (WLR), were obtained on SD-OCT images. Analysing SD-OCT data is more reasonable and accurate for detecting vascular structural changes caused by the long-term microcirculatory hypoxia and the stimulation of inflammatory factors in DR patients. For example, in the early stage of DR, when the diameter of retinal vessels remains unchanged, there is still a possibility of damage to endothelial cell function, leading to changes in WT and increases in the LD of blood vessels. However, the SD-OCT instrument cannot automatically locate the retinal region B, so, in this experiment, a transparent film marked with auxiliary lines was used to help the examiner locate that region (Figure 1(a)) to improve the operability and scientificity of the experiment.

In some early studies, it was found that DR development widened the retinal veins. However, due to technical limitations, it was difficult to quantitatively assess the specificity of such changes. This finding has not been listed as an observation indicator for DR at home or abroad, and only the relatively easily observed venous beading can be used as one of the markers for venous abnormalities and nonproliferative DR. In this study, it was found that the RVOD and RVLD of retinal veins in DR patients were larger than those in the NDR group, that WLR, WT, and WCSA in DR patients were much larger than those in non-DR patients, and that they were all linearly correlated with DR stage. The more severe the DR, the larger the diameters of the retinal vein and the thicker the vessel wall. However, there was no correlation between the duration of diabetes and retinal vessel diameter. In a recent development and validation study of a deep learning model involving more than 70,000 colour fundus photographs, researchers found that higher glycated-haemoglobin levels were associated with a wider CRVE, which is consistent with our findings [3]. After the occurrence of DR, if the retinal vein diameter is enlarged, this may predict the progression of DR. Clinically, it is generally accepted that patients with a long duration of diabetes and poorly controlled fasting blood glucose are more likely to develop DR and experience DR progression, reflecting the inseparable relationships between the diameter of retinal veins, fasting blood glucose level, and diabetes duration. It is certain that, in patients with T2DM, changes in retinal vascular diameter, especially venous diameter, are a warning sign for the occurrence and progression of microvascular complications in T2DM, and changes in venous diameter are the earliest microcirculatory abnormalities in patients with T2DM.

Although the likelihood of concurrent DR and severe progression increase with a longer diabetes duration, the diabetes duration is not completely equivalent to that of DR, and our multivariate linear regression analysis also excluded the correlation between diabetes duration and DR. This study also provides more detailed and practical reference indices of WT and WCSA. Because the impact of vessel pulsation on the vessel wall is less than the impact of vessel pulsation on vessel diameter, the measures such as WT and WCSA have higher reproducibility and higher research value. Some studies suggest that inflammatory response leads to venous distension and increases WT [10], further increasing WCSA, which is consistent with the results of this study. Moreover, retinal blood circulation disorders in diabetic patients often damage the function of vascular endothelial cells, raise the expression of nitric oxide in endothelial cells, mediate retinal flash stimulation through nitric oxide [11, 12], and thus participate in the regulatory process of vasodilatation. Some research also suggests that hyperglycaemia can cause retinal hypoxia and lactate accumulation, and venous dilation is a compensatory mechanism to increase blood supply to the retina [13]. Due to the limitations of the present experiments, the sensitivity of WT, RVOD, and RVLD and the timing of their changes could not be compared, though they should be in the future.

In this experiment, the RAOD, RALD, WT, and WCSA of retinal arteries in DR patients were significantly greater than those in non-DR patients. Moreover, RALD and WT were linearly correlated with DR stage. Cheung et al. [14] found that retinal arteries widened with increasing blood glucose and glycosylated haemoglobin and the progression of DR. Islam et al. [15] suggested that retinal artery diameter was not associated with the progression of DR, which was inconsistent with the above findings. However, some researchers have found that the retinal artery of DR patients became narrower [16]. Therefore, the correlations between diabetes and retinal artery remain controversial. This is perhaps due to the different races of the studied populations, the individual-level differences, and different sample sizes in different studies.

In patients with T2DM, fasting blood glucose level is a factor influencing the retinal vein diameter. When the fasting blood glucose level increases, the retinal vein diameter shows a tendency to dilate; however, the fasting blood glucose level does not affect the retinal artery diameter. This may be related to the structure and plasticity of the retinal artery and vein as well as the sensitivity of measurement methods. Clinically, retinal vein dilation is more likely to be observed when fasting blood glucose is elevated. In patients with T2DM, blood pressure influences the retinal artery diameter, and the retinal artery diameter tends to narrow with increasing blood pressure. Some researchers have given antihypertensive treatment to diabetic patients with hypertension to control their blood pressure to a good level and have found that the diameter of the retinal artery will gradually increase after treatment. The above conclusions suggest a strong correlation between retinal artery diameter and blood pressure.

In clinical practice, retinal artery stenosis is generally considered an early indicator of vascular damage due to age, hypertension, and other cardiovascular diseases, and structural changes to the retinal artery can provide an early warning about the health or disease of microvessels. In other words, if retinal arteriolar stenosis occurs in patients with T2DM, it may indicate high blood pressure, progression of diabetes, or vascular damage, suggesting the possibility of damage caused by complications such as DR and diabetic nephropathy [17, 18]. Strict blood pressure control can significantly reduce the macrovascular and microvascular complications in patients with T2DM. Moreover, it is easier to control the blood pressure stably than to control the blood glucose stably, and it is more effective to prevent and treat chronic complications of T2DM by controlling blood pressure than by controlling blood glucose. The conclusions drawn by recent studies on the changes in retinal artery diameter in diabetic patients are still controversial, and a larger sample size and further investigation are needed. However, we provide some new arterial parameters, such as WT and WCSA, in this study, and the intergroup differences between these measures were significant. These data have important research value and expand the ways in which the relationship between DR and retinal vessel diameters can be studied.

Wong et al. [19] showed that, in patients with T2DM, long-term stimulation to the vascular wall by factors such as hyperglycaemia, lactate accumulation, insulin resistance, and haemodynamic changes caused vascular wall remodelling. According to whether WCSA is enlarged or not, the remodelling can be divided into inward eutrophic remodelling and inward hypertrophic remodelling [20–23]. *In vitro* experiments by Endemann et al. [24] showed that eutrophic remodelling is a common form of structural change in resistance vessels in patients with essential hypertension, while hypertrophic remodelling is common in hypertensive patients with diabetes. In this study, the experimental results obtained through noninvasive SD-OCT on humans are consistent with the results of those *in vitro* experiments.

**Figure 2:**
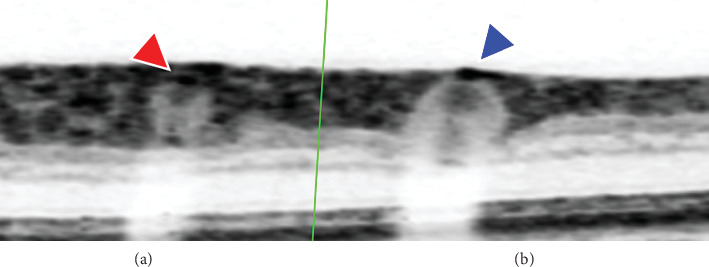
(a) The border of the retinal artery wall (red arrow) clearly identified. (b) The right retinal vein wall nearly overlaps with the retinal edge (blue arrow) and cannot be distinguished.

This study has several limitations. First, because the retinal vessels are close to the surface of the retina, it is difficult to distinguish the upper wall of the blood vessel from the tissue, resulting in a larger outer diameter of the blood vessel and a thicker vessel wall (Figure 2). Second, the FWHM method can reduce the measurement error. However, the method is not fully automated; there is a possibility of error, and the steps are very cumbersome and inefficient. Third, our sample size is small compared to other large-sample epidemiological studies. Fourth, dynamic changes occur in retinal blood vessel morphology, requiring long-term follow-up. At present, we are jointly developing retinal blood vessel measurement software based on deep learning, which can increase the accuracy of blood vessel mass measurement, thus improving the database.

## 5. Conclusion

The LDs and ODs of retinal arteries and veins in patients with DR are widened, the vessel wall is thickened, and the cross-sectional area of the vessel wall is increased. The LDs and ODs of retinal veins and vessel WT in patients with DR are linearly correlated with DR stage and gradually widen with the progression of DR. Changes in the diameters of retinal arteries and veins in patients with DR are not correlated with the duration of diabetes.

## Figures and Tables

**Table 1:** General clinical characteristics of patients in the two groups.

	Number of cases	Sex, male (female)	Age	Body mass index
NDR group	75	39 (36)	62.40 ± 10.05	25.26 ± 3.32
DR group	65	43 (22)	65.50 ± 8.41	24.30 ± 3.32
*P* value		0.090	0.051	0.092
*χ * ^2^ or *t* value		2.875	−1.965	1.699

*Note*. Body mass index and age were analysed by the independent-samples *t*-test, and sex was analysed by the *χ*^2^ test.

**Table 2:** Parameters of retinal arteries in the NDR and DR groups.

	RALD (*μ*m)	RAOD (*μ*m)	WLR (*μ*m)	WT (*μ*m)	WCSA (*μ*m^2^)
NDR group	104.88 ± 15.68	135.60 ± 17.22	0.29 ± 0.06	15.26 ± 2.56	5824.64 ± 1330.07
DR group	128.80 ± 36.00	147.01 ± 17.55	0.28 ± 0.05	18.29 ± 5.97	6647.51 ± 1590.52
*P* value	<0.001	<0.001	0.081	<0.001	<0.001
*t* value	−5.214	−3.872	1.756	−3.998	−3.333

**Table 3:** Parameters of retinal veins in the NDR and DR groups.

	RVLD (*μ*m)	RVOD (*μ*m)	WLR (*μ*m)	WT (*μ*m)	WCSA (*μ*m^2^)
NDR group	133.66 ± 21.07	156.43 ± 23.36	0.17 ± 0.03	11.38 ± 2.33	5321.03 ± 1695.19
DR group	146.17 ± 34.42	180.20 ± 38.70	0.23 ± 0.05	17.01 ± 4.23	8962.13 ± 3897.34
*P* value	0.012	<0.001	<0.001	<0.001	<0.001
*t* value	−2.545	−4.313	−7.827	−9.529	−6.982

**Table 4:** Correlations between retinal artery-associated factors.

		RALD	RAOD	WT	WLR	WCSA
Age	*r* value	0.031	−0.013	0.161	0.271	0.125
	*P* value	0.807	0.915	0.200	0.029	0.320

DR stage	*r* value	0.480	−0.139	0.524	0.142	−0.063
	*P* value	<0.001	0.269	<0.001	0.259	0.619

Diabetes duration	*r* value	−0.087	0.047	−0.184	−0.136	−0.026
	*P* value	0.489	0.708	0.142	0.281	0.835

**Table 5:** Correlations between retinal vein-associated factors.

		RVLD	RVOD	WT	WLR	WCSA
Age	*r* value	0.052	0.074	0.127	0.114	0.107
	*P* value	0.679	0.556	0.312	0.367	0.395

DR stage	*r* value	0.588	0.603	0.369	−0.090	0.567
	*P* value	<0.001	<0.001	0.003	0.474	<0.001

Diabetes duration	*r* value	−0.075	−0.017	0.226	0.280	0.092
	*P* value	0.552	0.891	0.070	0.024	0.464

**Table 6:** Correlations between retinal artery-associated factors (multivariate regression).

Equation	*R * ^2^	*F*	*b*	*P* value
RALD = 82.963 + 17.633 × DR stage	0.231	18.908	17.633	<0.001
WT = 9.997 + 3.193 × DR stage	0.274	23.817	3.193	<0.001
WLR = 0.162 + 0.002 × age	0.074	5.006	0.002	0.029

**Table 7:** Correlations between retinal vein-associated factors (multivariate regression).

Equation	*R * ^2^	*F*	*b*	*P* value
RVLD = 92.532 + 20.632 × DR stage	0.346	18.908	33.287	<0.001
RVOD = 118.273 + 23.819 × DR stage	0.364	36.012	23.819	<0.001
WT = 12.871 + 1.593 × DR stage	0.136	9.916	1.593	0.003
WLR = 0.208 + 0.004 × diabetes duration	0.078	5.344	0.004	0.024
WCSA = 3108.217 + 2251.506 × DR stage	0.321	29.814	2251.506	<0.001
