# Corrigendum to “Application of the Full-Width-at-Half-Maximum Image Segmentation Method to Analyse Retinal Vascular Changes in Patients with Diabetic Retinopathy”

**DOI:** 10.1155/johe/9861613

**Published:** 2025-09-03

B. L. Xu, Y. J. Li, W. L. Zhou, H. J. Zhan, J. Y. Lu, and Y. H. Tong, “Application of the Full-Width-at-Half-Maximum Image Segmentation Method to Analyse Retinal Vascular Changes in Patients with Diabetic Retinopathy,” *Journal of Healthcare Engineering* 2022 (2022): 6726499, https://doi.org/10.1155/2022/6726499.

The article was originally published incorrectly as a Review article. The correct article type is Research Article, and the article has been updated to reflect this.

We apologize for this error.

